# Intestinal Fibrosis in Inflammatory Bowel Disease and the Prospects of Mesenchymal Stem Cell Therapy

**DOI:** 10.3389/fimmu.2022.835005

**Published:** 2022-03-18

**Authors:** Yifei Wang, Bin Huang, Tao Jin, Dickson Kofi Wiredu Ocansey, Jiajia Jiang, Fei Mao

**Affiliations:** ^1^Aoyang Institute of Cancer, Affiliated Aoyang Hospital of Jiangsu University, Suzhou, China; ^2^Key Laboratory of Medical Science and Laboratory Medicine of Jiangsu Province, School of Medicine, Jiangsu University, Zhenjiang, China; ^3^General Surgery Department, Affiliated Aoyang Hospital of Jiangsu University, Suzhou, China; ^4^Department of Gastrointestinal and Endoscopy, The Affiliated Yixing Hospital of Jiangsu University, Yixing, China; ^5^Directorate of University Health Services, University of Cape Coast, Cape Coast, Ghana

**Keywords:** intestinal fibrosis, IBD, mechanism, MSC, therapy, immune cells

## Abstract

Intestinal fibrosis is an important complication of inflammatory bowel disease (IBD). In the course of the development of fibrosis, certain parts of the intestine become narrowed, significantly destroying the structure and function of the intestine and affecting the quality of life of patients. Chronic inflammation is an important initiating factor of fibrosis. Unfortunately, the existing anti-inflammatory drugs cannot effectively prevent and alleviate fibrosis, and there is no effective anti-fibrotic drug, which makes surgical treatment the mainstream treatment for intestinal fibrosis and stenosis. Mesenchymal stem cells (MSCs) are capable of tissue regeneration and repair through their self-differentiation, secretion of cytokines, and secretion of extracellular vesicles. MSCs have been shown to play an important therapeutic role in the fibrosis of many organs. However, the role of MSC in intestinal fibrosis largely remained unexplored. This review summarizes the mechanism of intestinal fibrosis, including the role of immune cells, TGF-β, and the gut microbiome and metabolites. Available treatment options for fibrosis, particularly, MSCs are also discussed.

## Introduction

Intestinal fibrosis is a common complication of IBD and is usually defined as an excessive accumulation of scar tissue in the intestinal wall. Intestinal fibrosis can occur in both forms of IBD: ulcerative colitis (UC) and Crohn’s disease (CD), but mostly in CD (1). It represents a challenge for both basic scientists and clinicians, with respect to diagnosis, pathogenic mechanisms, and clinical management, owing to the lack of reliable and easily transferrable experimental models of fibrosis, the lack of drugs targeting fibrosis, and the scarcity of predictive markers (2). In the past years, researchers have intensely explored the mechanism of fibrosis. Similar to fibrosis of other organs, intestinal fibrosis can activate an immune response, release cytokines, and act on various cells of the intestine, including epithelial cells, fibroblasts, and smooth muscle cells, accelerating the accumulation of extracellular matrix and depositing collagen. The common treatment for intestinal fibrosis is always around anti-inflammation but not directly anti-fibrosis. Although existing treatment has some preventive effects on fibrosis, it still does not prevent recurrence (3). In the existing clinical data, there is an indication that many patients with IBD develop the later stage of stenosis, and mostly experience unsatisfactory surgical solutions (4).

In recent years, MSCs therapy has become a popular central issue in anti-tumor and tissue regeneration because of their self-differentiation ability, the release of regulatory factors, and particularly, the secretion of extracellular vesicle (EV) (5). Many studies have found that MSCs can play an important role in the fibrosis of various organs. Notwithstanding, there has not been much elaboration on the role of MSCs in intestinal fibrosis. In addition, few studies have confirmed the role of MSCs in the treatment of IBD (6). There is prospect and confidence that MSCs could be an effective therapeutic solution to intestinal fibrosis. We, therefore, explore the mechanism of intestinal fibrosis and discuss available treatment options, particularly the prospects of MSCs in IBD treatment.

## Intestinal Fibrosis

IBD, a chronic inflammatory disease that includes CD and UC, severely affects the quality of life of the patient (7). A lot of factors such as environment, genetic, gut microbiome, and immune disorder can affect the occurrence and progression of IBD (8, 9). In addition to affecting the quality of life of patients, IBD also increases the risk of colorectal cancer between 1.4 to 2.2 fold, with decreased survival rate in patients with IBD (10).

As a crucial complication of IBD, intestinal fibrosis serves as a common and great challenge for IBD therapeutic. It is documented that fibrotic complications occur in more than 50% of patients with CD, mainly reflected as stricture and penetrate. Stricture is a serious problem with an 8% incidence in UC, whereas fibrostenotic complications lead to stricture formation (narrowing), intestinal obstruction, and a need for surgical intervention, and as such, is one of the largest unresolved clinical challenges in IBD (11). The development of intestinal fibrosis is complex and the specific mechanism has not been understood until today, however, researchers widely believe that the progression of intestinal fibrosis includes the following steps: cells injury, production of transforming growth factor (TGF-β1), recruitment of inflammatory cells, and activation of myofibroblasts and collagen-producing cells (12–16).

Currently, there is no anti-inflammation medicine that effectively prevents the development of intestinal fibrosis. Patients with IBD always undergo surgery under the help of endoscopic when strictures occur (17–19). Therefore, the discovery of therapy targeting intestinal fibrosis reverse or prevention will be a big breakthrough in medicine. One of the keys focused in the study of colon fibrosis is the hope to detect the appearance of fibrosis earlier in patients. Apart from the help of colonoscopy and endoscope, researchers are probing at the molecular level, including serum genetic markers (20), extracellular matrix (ECM) components (21), growth factors (22), and miRNAs (23) in fibrosis.

**Figure 1:**
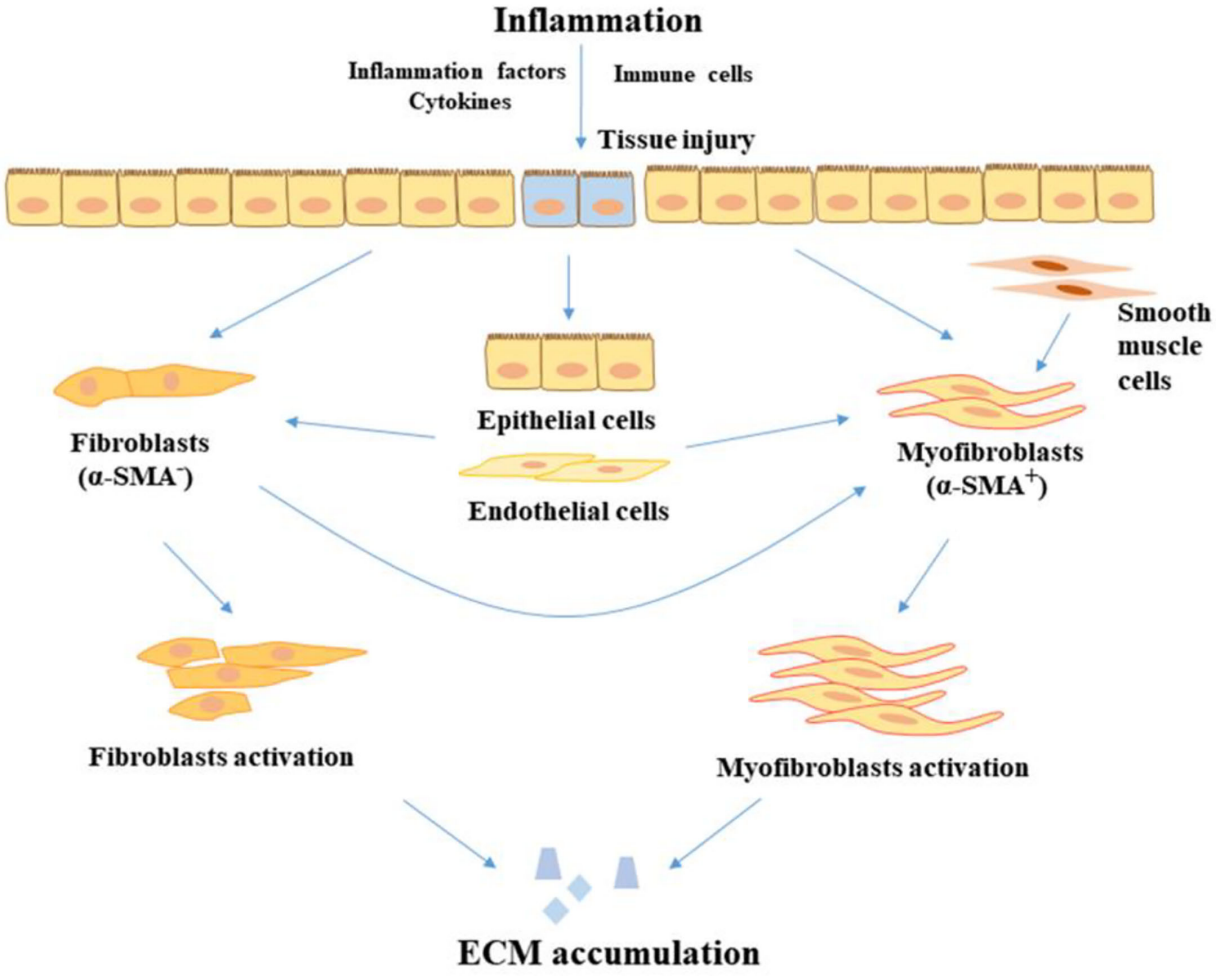
The progression of fibrosis in IBD. In the inflammatory condition, tissue injury is stimulated by inflammatory factors and cytokines, leading to the proliferation and activation of fibroblasts in the intestine. In addition, epithelial cells and endothelial cells transition to mesenchymal cells. Fibroblasts and smooth muscle cells also transition to myofibroblasts. Myofibroblasts and fibroblasts accumulate the product of ECM, leading to the development of fibrosis. ECM, extracellular matrix; α-SMA, alpha-smooth muscle actin.

It is normally considered that unhealed inflammation triggers the excessive accumulation of ECM and increased production of collagen, indicating that the occurrence and severity of colon fibrosis may show a positive correlation to an inflammatory condition **Figure 1**. A study found that several fibrosis mediators including the TGF-β signaling pathways, pro-fibrotic cytokines, and other fibrosis-related factors were increased even in healed mucosal of UC patients (24). Moreover, an earlier study also found that intestinal fibrosis develops despite the removal of an inflammatory stimulus and elimination of inflammation. This implicates that, early intervention ameliorates but does not abolish subsequent fibrosis, suggesting that fibrosis, once initiated, is self-propagating, therefore a very early top-down interventional approach may have the most impact on fibrostenosing diseases (25). Similarly, Hünerwadel and colleagues found that severity of inflammation does not affect fibrosis, by using established animal models from IL10^-/-^ mouse (26). These observations clearly show the current situation, whereby existing anti-inflammation drugs exert an insufficient anti-fibrotic effect in the therapeutic process. Otherwise, the use of current drugs is hard to prevent the appearance of fibrosis and stricture recurrence (27). Therefore, it is a great challenge to understand the development of intestinal fibrosis and explore effective therapeutic and diagnostic methods that could finally help ease patients’ care challenges.

## Mechanism of Intestinal Fibrosis

### Role of MMPs and TIMPs

Intestinal fibrosis followed by chronic and recurrent inflammation leads to deposition of extracellular matrix (ECM) in the mucosa, including collagen and fibronectin. The accumulation of ECM can be regulated by one pair of proteins, matrix metalloproteinases (MMP) and their inhibitors, tissue inhibitors metalloproteinases (TIMP) (28). MMPs regulate fibrosis by degrading the ECM that is normally deposited as the tissue renews. However, MMPs’ function is tightly regulated by TIMPs, which inhibit MMP activity in a 1:1 ratio. Fibrotic tissue resected from IBD patients and pre-clinical models of intestinal fibrosis shows altered expression of MMP-2, MMP-3, MMP-8, MMP-9, and TIMP-1 to varying degrees (28, 29). However, MMPs and TIMPs are also increased in inflamed intestinal tissue and it remains unclear how their expression is altered in inflamed relative to fibrotic tissue (30).

### The Role of TGF-β Signaling

Transforming growth factor-β (TGF-β) plays an important role in inflammation, cell proliferation, and cancer. There are three subtypes of TGF-β; TGF-β1, TGF-β2, and TGF-β3. It is known to activate downstream mediators like Smad2, Smad3, and Smad7 to play a positive or negative regulatory role. *In vivo*, it does not only regulate proliferation to maintain homeostasis but also promotes the development of cancer and fibrosis (31). A clinical trial showed that the application of anti-TGF-β is able to reduce fibronectin and high molecular weight type IV collagen production (32). The use of TNFα antagonists can decrease the occurrence of fibrosis on patients after irradiating through lower TGF-β (33). Moreover, an earlier study proved that urinary TGF-β is a potential marker and predictor of hepatocellular carcinoma (HCC) (34).

**Figure 2:**
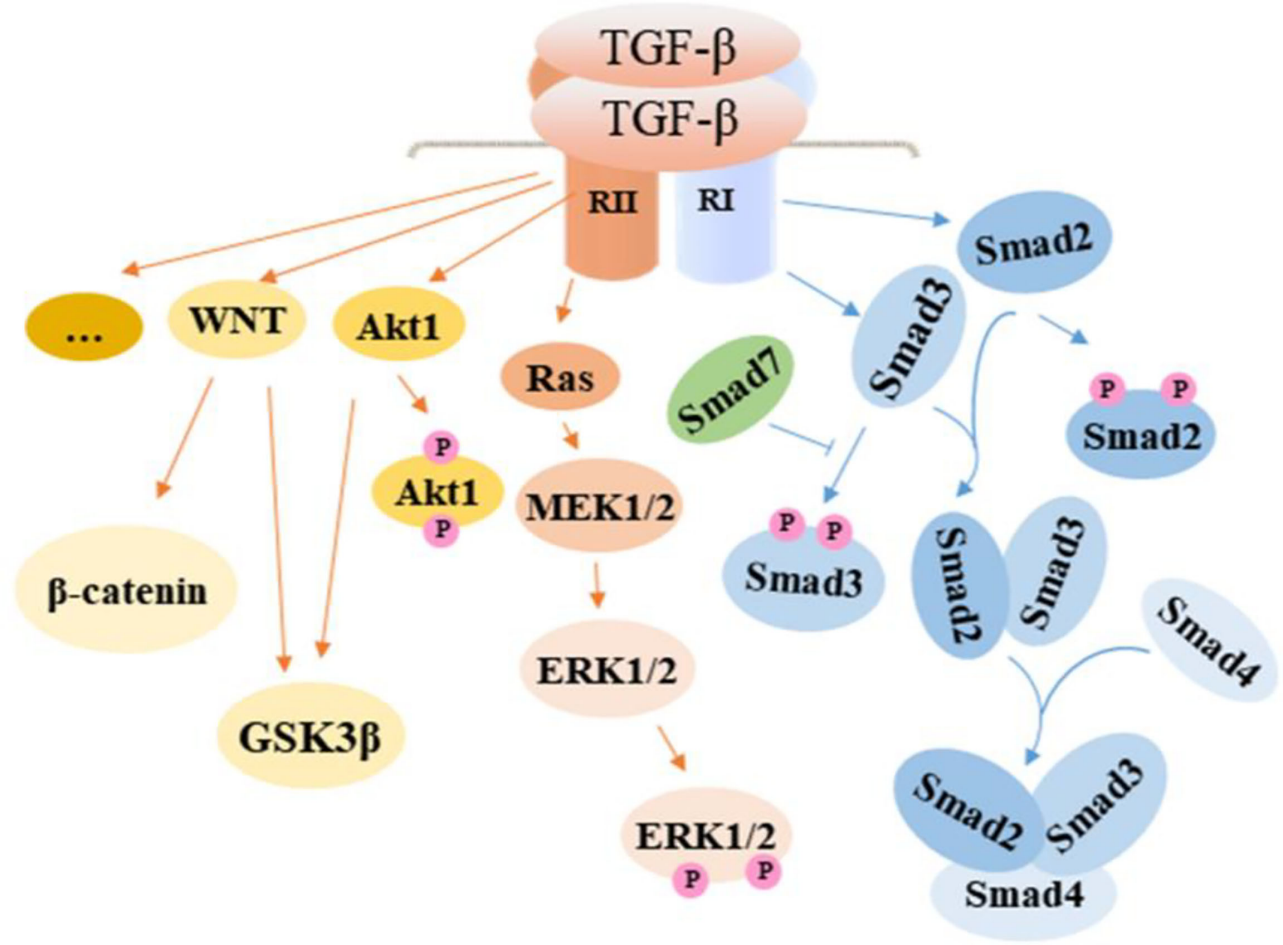
TGF-β activated signaling pathway in intestinal fibrosis. TGF-β promotes fibrosis by regulating related cells through the activation of the canonical Smad signaling pathway and noncanonical pathway including MAPK and WNT, which contribute to the development of EMT in epithelial cells, the proliferation of fibroblasts, and transformation of fibroblasts and smooth muscle cells to myofibroblasts. In effect, ECM is overexpressed, resulting in increased collagen deposition. TGF-β, transforming growth factor β; Smad, drosophila mothers against decapentaplegic; Ras, rat sarcoma; MEK, methyl ethyl ketone; ERK, extracellular regulated protein kinases; GSK3β, glycogen synthase kinase3β.

As a central cytokine in the development of intestinal fibrosis, TGF-β can play a role as an upstream molecule to activate downstream signaling pathways. TGF-β binds receptors to activate the sphingosine kinase 1/sphingosine-1-phosphate/mammalian target of rapamycin (SPHK1/S1P/mTOR) pathway and accelerates the production of pro-fibrotic molecules, which finally contribute to the occurrence of intestinal fibrosis (35). The TGF-β/Smad signaling pathway is seen as a vital signaling pathway in the development of fibrosis in a number of organs. This pathway can regulate myofibroblast proliferation, fibroblast transition to myofibroblast, and the process of epithelial-to-mesenchymal transition (EMT). In addition to the canonical TGF-β/Smad signaling pathway, TGF-β can activate other signaling pathways, including extracellular regulated protein kinases (ERK) signaling pathway, Phosphatidylinositol-3-kinase/protein kinase B (PI3K/AKT) signaling pathway, and WNT signaling pathway **Figure 2**.

In addition to its target on cells through signaling pathways, TGF-β seems to associate with other physiological changes in intestinal fibrosis. Reactive oxygen species (ROS) can lead to the development of intestinal fibrosis on the account of TGF-β dependency. NF-E2-Related Factor 2 (Nrf2) is a nuclear transcription factor that plays a role in defending against oxidative stress in cells. A study found that Nrf2 could suppress intestinal fibrosis *in vivo* and *in vitro*. In that study, TNBS-induced-fibrosis mice were given Nrf2 agonist, which resulted in a reduced degree of fibrosis compared with the no agonist group. Similarly, the application of siNrf2 inhibited the differentiation of TGF-β-induced CCD18Co cells (36).

Due to its critical role in the development of fibrosis, TGF-β targeted inhibition has been seen as a worth considering therapy approach in intestinal fibrosis. In exploring this option, peroxisome proliferator-activated receptor γ (PPARγ), which is a member of ligand-activated transcription factors of the nuclear hormone receptor superfamily and involved in many diseases including inflammation has been tested. The result showed that PPARγ regulator GED-0507-34 Levo could ameliorate inflammation-related fibrosis in the colon. Oral gavage of the TGF-β inhibitor daily in DSS fibrotic mice effectively decreased the expression of fibrosis markers in the colon. Similarly, *in vitro*, GED could inhibit the differentiation of myofibroblasts under the stimulation of TGF-β, hence repressing the process of EMT of HT29, a type of colon epithelial cells, and the expression of fibrosis marker in human primary fibroblast (37).

### The Contribution of Immune Cells and Their Cytokines

#### T Cells

T cells are one of the most important immune cells in IBD. Studies showed T cells can regulate fibrosis in different tissues **Table 1**. The regulatory mechanism of T cells in intestinal cells has not been well documented. Several pieces of research demonstrate that T cells subsets such as Th1, Th2, Th9, Th17, Th22, and regulatory T cells (Treg), and their expressed cytokines could promote the development of intestinal fibrosis.

**Table 1:** Function of T cells subsets in different fibrosis.

Immune cells	Organ	Effects in fibrosis	References
Th1 cells	Lung	Anti-fibrotic	(38)
	Heart	Pro-fibrotic	(39, 40)
	Liver	Anti-fibrotic	(41)
Th2 cells	Skin	Pro-fibrotic	(42)
	Liver	Pro-fibrotic	(41, 43)
	Kidney	Pro-fibrotic	(44)
	Biliary	Pro-fibrotic	(45)
Th9 cells	Liver	Pro-fibrotic	(46)
Th17 cells	Liver	Pro-fibrotic	(41)
	Lung	Pro-fibrotic	(38)
	Heart	Pro-fibrotic	(47)
Th22 cells	Liver	Anti-fibrotic	(48)
Treg cells	Lung	Anti-fibrotic	(49)
	Lung	Pro-fibrotic	(50)
	Kidney	Pro-fibrotic	(51)
Cytotoxic T Cell	Lung	Pro-fibrotic	(52)
	Kidney	Anti-fibrotic	(53)
	Thyroid	Pro-fibrotic	(54)
NKT cells	Liver	Anti-fibrotic	(55)
	Liver	Pro-fibrotic	(56)
	Heart	Anti-fibrotic	(57)

Th17 is one type of T helper cell that mainly produces interleukin-17 (IL-17). The IL-17 produced by Th17 cells and innate lymphoid cells has been confirmed to play an important role in IBD (58, 59). Recently, researchers found that it also exhibits a crucial function in intestinal fibrosis by contributing to its pathogenesis. Jian Li and colleagues investigated the level of profibrotic molecules and collagen in the blood of Balb/c mice and found that the group treated with anti-IL-17 significantly decreased the quantity of collagen and expression of pro-fibrogenic molecules, leading to alleviated intestinal fibrosis (60). At the same time, as a member of IL-17, IL-17A is found to increase intestinal epithelial cell-6 (IEC-6) and potentially induce EMT through reducing E-cadherin expression and increasing the expression of Vimentin (61). Contrary to these reports, a study found that Tregs and IL-17 had no important contributions in regulating the DSS-induced fibrosis model (62). Most of the studies on the function of IL-17/Th17 have been conducted using an animal model, which could not completely imitate the real situation in humans, therefore, more studies are needed to confirm these observations.

Th2 is another T helper cell that produces IL-4, IL-5. and IL-13. IL-13 is able to inhibit MMP production, causing elevated ECM deposition and induced TGF-β function (63). Interleukin-10 (IL-10) is an anti-inflammation cytokine and has been seen as a potential and novel target in anti-fibrotic therapies. IL-10 is an anti-inflammation cytokine and has been seen as a potential and novel target in anti-fibrotic therapies. IL-10 is first found as the product of Th2 cells in the process of inhibiting T helper 1 cell and it has been confirmed to be produced by different immune cells (macrophage, B cells, dendritic cells, mast cells, and others) (64). IL-10 plays an important role in inhibiting fibrosis. A study found that IL-10KO mice treated with IL-10 decreased the expression of collagen I and TGF-β (65). In addition, compared with wild-type mice, IL-10 mice showed higher fibrosis scores under the treatment (66).

Tregs are a subset of T cells and secret IL-10 which functions as an anti-inflammatory agent. Contrary to other subtypes of T cells, Tregs seem to be activated in the process of anti-fibrosis. A study found that treatment that induces Tregs could effectively ameliorate intestinal fibrosis in mice (67).

IL-12 is a product that affects the polarization of naïve T helper cells to the Th1 phenotype, while IL-23 plays an important role in stabilizing Th17. It is reported that the administration of p40, which blocks IL12 and IL 23 in TNBS chronic colitis animals, could efficiently relieve the deposition of collagen (68). In another study, the researchers used Th-related cytokine to induce colon fibroblast and found that the cytokines could up-regulate or down-regulate pro-fibrotic gene expression (69). This affirms the crucial role of T cells and related cytokines in the development of intestinal fibrosis. In addition, apart from the commonly used chemical damage model, researchers usually use the T cells transfer model to study T cell-related regulatory factors in inflammation (70). This model also serves as an appropriate medium for discussing T cell-related effects on fibrosis.

#### Macrophages

Macrophages are a critical part of the immune response in IBD. A recent study showed that macrophage in patients’ blood has the potential to differentiate IBD patients into different groups with different phenotypes and may therefore help determine response to therapy (71). In recent years, several studies have focused on the role of macrophages in intestinal fibrosis. Intestinal macrophages keep gut homeostasis through secreting several cytokines, regulating molecules, and participating in epithelial proliferation (72). The disorder of macrophage leads to aberrant repair, abnormal inflammatory mediator and growth factor production, and altered communication between macrophages and fibroblasts, epithelial cells, and endothelial cells, finally promoting the progression of fibrosis (73). Macrophages are divided into M1 macrophages and M2 macrophages. In the inflammatory environment, M1 releases pro-inflammatory cytokines while M2 releases IL-10 and TGF-β, which inhibit inflammation (74). Although M2 macrophages have anti-inflammatory properties, it does not mean that they automatically play an anti-fibrotic role. Both M1 and M2 macrophages are involved in the occurrence and development of fibrosis (75). Notwithstanding, the different macrophage phenotypes play different roles in fibrosis. Pedro Salvador and colleagues found that CD16+ macrophages are increased in the mucosa of CD patients and were mediated by signal transducer and activator of transcription-6 (STAT6), where the deficiency of STAT6 elevated the population of CD16+ macrophages (76). There are relatively few studies on the role of macrophages in intestinal fibrosis, providing a research gap to be explored in the future.

#### Mast Cells

Mast cells (MCs) are innate immune cells capable of responding to different stimuli. They are fundamental elements of the intestinal barrier as they regulate epithelial function and integrity, modulate both innate and adaptive mucosal immunity, and maintain neuro-immune interactions, which are closely linked to the functioning of the gut (77–79). Although the role of MCs, which are members of the sentinel immune cell population, remains largely unknown in intestinal fibrosis, there are reports of a large influx of MCs into the muscularis externa of the small intestine in fibrosis (80), and the promotion of intestinal fibrosis after the breakdown of the mucosal barrier (81).

The role of MCs in intestinal inflammation appears to own two sides, i.e., anti-inflammation and inflammation. On the anti-inflammation side, a study showed that MCs alleviate colitis and (82) protect against intestinal barrier injury in IL-10 deficient mouse models. Similarly, the infiltration of MCs in the intestine tissue is especially increased at the DSS-induced experimental colitis remission phase and deficiency of MCs could lead to failed tissue repair (83). On the contrary, other studies have demonstrated the inflammatory property, reporting that MCs accelerate the development of intestinal inflammation. For example, Musheng Li and colleagues examined MCs-derived exosomes, which showed that exosomal miR-223 from human mast cells-1 (HMCs-1) could destroy intestinal epithelial function through inhibiting the expression of CLDN8 (Claudin 8) in epithelial cells (84). These findings implicate that MCs may have a more important role in chronic inflammation, even in fibrosis development (85).

MCs are confirmed to play a significant role in tissue fibrosis of several organs, including liver, lung, pulmonary, and atrial (86, 87). MCs also regulate fibrosis in Chronic Graft-Versus-Host Disease. Interestingly, MCs seem to show contradictory effects in different conditions from different researches. Some studies report that when fibrosis starts in the tissue, the MCs increase and are activated, as shown by degranulation and secretion of cytokines including TGF-β and other tryptases, which accelerate the ECM production and promote the progression of fibrosis (88). Bin Liu and colleagues also documented the elevated presence of MCs in fibrotic intestinal tissues, and MCs affected the development of fibrosis with the release of tryptase. Unfortunately, researchers in this area are not rich and await further exploration (89).

### EMT, EndoMT, and Intestinal Fibrosis

EMT is an important pathophysiological process in the occurrence and development of many conditions, including intestinal fibrosis and cancer initiation, invasion, and metastasis (90). EMT is an important hallmark of intestinal fibrogenesis through which epithelial cells lose their polarity or their epithelial phenotype and transform into mesenchymal cells functionally and morphologically (91). EMT-associated molecules were found in the fibrotic lesion of CD patients (77). According to Dolores Ortiz-Masiá and colleagues, the process of EMT is accompanied by fistula development, which is an abnormal tract between two epithelial cells and is associated with fibrosis (92). Certain pro-inflammatory cytokines such as IL-17A have been shown to possess profibrotic properties as they are associated with fibrosis of multiple organs including the intestine. IL-17A is found to participate in the initiation and development of intestinal fibrosis through inducing EMT (61).

Other mechanisms involved in EMT induction that leads to intestinal fibrosis include toll-like receptor 4 (TLR4) and succinate stimulation. The absence of the TLR4 gene attenuated chronic inflammation and colonic macrophages infiltration and suppressed intestinal fibrosis and collagen deposition. Moreover, suppression of TLR4 transcription affected myofibroblasts’ activity, collagen synthesis, and EMT in CCD-18Co cells, a human cancer cell line (93). In another study, succinate and its receptor UCNR1 were up-regulated around CD-fistulas and activated WNT signaling-mediated EMT in intestinal epithelial cells (92). In addition to studies of EMT in intestinal fibrosis in cell lines and animal models, manner-organoid, a novel 3D model in colon studies has been shown to be more specific than animal models and more complex than cell models. In this model, Soojung Hahn and colleagues used TGF-β or TNF-α to stimulate the organoid colon model and the result showed a combination of the two cytokines could effectively promote the expression of mesenchymal markers like N-cadherin and fibrotic-related factors including α-SMA (94).

In recent years, in addition to EMT, endothelial-to-mesenchymal transition (EndoMT) has also been reported as a novel mechanism in fibrosis, where transcription factors involved in the transformation process were confirmed in both inflamed human and murine intestine (95, 96). Moreover, EndoMT has been detected in experimental colonic fibrosis of Tie2- green fluorescent protein (GFP) reporter-expressing mice (97).

### Involvement of the Gut Microbiome in Intestinal Fibrosis

The gut microbiome influences health and disease. Changes in the composition of the gut microflora, immune system, or intestinal barrier function can upset the host-microbiome interaction and lead to inflammation and fibrosis (98). For instance, adherent-invasive Escherichia. coli (AIEC), a type of gut bacteria, is confirmed to be involved in IBD, especially CD (99, 100). A study discovered that AIEC could colonize the intestine when acute inflammation occurs, leading to fibrosis *via* increased expression of ST2 -the receptor of IL-33, under the mediation of Flagellin (101). The microbial infection triggers a disorder of the immune microenvironment, where the persistent infection of AIEC leads to an active T-helper 17 response and increases the fibrotic growth factors (102). Tumor necrosis factor-like cytokine 1A (TL1A, TNFSF15) is associated with IBD, regulating the location and severity of intestinal inflammation and fibrosis. TL1A production is elevated in the inflamed gut mucosa, is linked with fibrostenosing CD, and is well dependent on the gut microbiome. The authors showed that pro-fibrotic and inflammatory phenotype resulting from TL1A-upregulation was abrogated in the absence of resident microbiota (8).

Salmonella enterica serovar Typhimurium also plays a key role in intestinal fibrogenesis. Katrin Ehrhardt and colleagues found that mice develop intestinal fibrosis under persistent infection with Salmonella enterica serovar Typhimurium through inducing protease expression in macrophages and epithelial cells (103). Microbial products such as cell wall components can be directly pro-fibrogenic, while the administration of fecal material or anaerobic bacteria into autologous animals could also trigger intestinal fibrosis (98). Generally, the CD is associated with shifts in the composition of the enteric microbiota, with overall reduced bacteria diversity and significantly depleted abundance of the phyla Firmicutes and Bacteroidetes, while Proteobacteria and Actinobacteria increase (104).

However, the gut microbe does not only contribute to the induction but also the prevention of intestinal fibrosis. The gut microbe and related proteins can protect the intestine from fibrotic injury. For example, a study found heat-shock-protein 56 (HSP56) secreted by invasive Lactococcus lactis can reduce the severity of fibrosis (105).

When the microbiome leads to dysregulation of the immune response, the immune and non-immune cells sense the pathogen-associated molecular patterns (PAMPs) from microbe with the help of pattern recognition receptors (PRRs), which consists of TLRs and Nod-like receptors (NLRs). MyD88 is an adaptor molecule that helps all TLRs except TLR3 to release signals, hence, several researchers believe that MyD88 may have a role in intestinal fibrosis. Shuai Zhao and colleagues confirmed that intestinal fibrosis improves when the gene of MyD88 in α-SMA positive cells is deleted (106). However, an earlier study from C. Lutz et al. indicated that MyD88 shows no or little effect in intestinal fibrosis (107). As a member of TLRs, TLR4 is reported to participate in intestinal fibrosis. Studies show that intestinal fibrosis and the deposition of collagen are suppressed under the silence of the TLR4 gene *in vivo* and *in vitro* (93).

### Autophagy and Intestinal Fibrosis

Autophagy is an evolutionarily conserved important process for the turnover of intracellular substances in eukaryotes with cytoplasmic cargo transferring to the lysosome and degradation (108). Autophagy is considered to be widely involved in various conditions such as intestinal fibrosis (109), cancer (110), kidney diseases (111), and pulmonary diseases (112).In intestinal epithelial cells of IBD subjects, autophagy could regulate programmed cell death and limit the development of colitis (113). At the same time, autophagy functions in regulating inflammatory cytokines, such as IL-1β (114). Under physiological conditions, the system of cellular adaptation permits the intestinal mucosa to maintain the gut barrier function and avoids excessive immune response to non-self-antigens from commensal microbes or dietary origin (115, 116). Interestingly, autophagy inhibits the pathogenic immune response to dietary antigens in cystic fibrosis, an inherited disorder that causes severe damage to the digestive system, lungs, and other organs (115).

A study confirmed that autophagy played an important role in regulating intestinal fibrosis in mice, where the administration of autophagy inhibitor, resulted in the appearance of intestinal fibrosis, implicating autophagy as a protective mechanism against fibrosis generation (64). Moreover, autophagy is reported to increase in the mice colitis model, which helps to ease inflammation (3). In another study, the antifibrotic effects of curcumin were demonstrated *via* its alleviation of IL-6-induced endothelial-to-mesenchymal transition through promoting autophagy in allografted organs and human umbilical vein endothelial cells (HUVECs) (117). However, it is reported that autophagy in immune cells could induce an immune response that finally aggravates fibrosis. The autophagy in CX3Cr1+ mononuclear phagocytes could up-regulate IL-23/IL-22 axis (15).

### The Link of the Renin-Angiotensin System (RAS) to Intestinal Fibrosis

The renin-angiotensin system (RAS) is widely present in various parts of the body including blood vessels, kidneys, and heart, and is an important regulatory system for several disease conditions. Recent researches confirm that the renin-angiotensin-aldosterone system (RAAS) interacts with the TGF-β pathway, participating in fibrosis development by regulating cells and cytokines. Further exploration revealed that different pathways of RAAS may lead to different outcomes; while some molecules prevent fibrosis, others promote it (118).

Other studies show that the imbalance of RAS induces inflammation and fibrosis in the colon. For example, a recent study by Garg et.al., reported that Ang (1-7) reduced the proliferation of myofibroblasts and secretion of collagen, whereas Ang II promoted these events. When the quantity of RAS components in IBD patients was compared with healthy people, circulating renin and alternative RAS components were high in IBD patients. Interestingly, patients with CD had reduced the requirement of hospitalization and surgery after treatment with RAS blockers (119). These findings indicate that drugs targeting the RAS, besides being antihypertensive, also possess antifibrotic and anti-inflammatory properties and could offer an inexpensive alternative to control inflammation and fibrosis in the gut.

### The Role of Non-Coding RNAs in Fibrosis

In recent studies, the importance of non-coding RNAs (ncRNAs) stands out in fibrotic diseases in that ncRNAs exhibit a remarkable variety of biological functions in modulating fibrogenic responses. The participation of ncRNAs in intestinal fibrogenesis makes them potential therapeutic targets and diagnostic biomarkers in the management of intestinal fibrosis (120). The overexpression of certain microRNA(miRNAs) can inhibit the development of fibrosis. For instance, studies on MiR-200 in intestinal fibrosis show down-regulated expression of miR-200 family in intestine tissue from CD patients (121, 122). Moreover, treatment of IEC-6 with micro-vesicles carrying miR-200b induced by TGF-β prevented the process of EMT and alleviated fibrosis (123). In radiation-induced intestinal fibrosis, lncRNA WWC2-AS1 functions as a competing endogenous RNA in the regulation of FGF2 expression *via* sponging miR-16. The resultant inhibition of FGF2 function, prevents intestinal cell proliferation, migration, invasion, and fibrosis (124). Contrary to these observations, other studies have reported the fibrosis-promoting effects of ncRNAs. For example, it is documented that the miR-29 family enhances collagen deposition (125).

In addition to the intestine, several studies have demonstrated ncRNAs to participate in the fibrotic diseases of multiple organs including liver diseases, myocardial fibrosis, and renal fibrosis. The ncRNAs involved in fibrotic diseases mainly consist of miRNAs, long noncoding RNAs (lncRNAs), and circular RNAs (circRNAs). ncRNAs modulate the function of mesenchymal cells, inflammatory cascades, ECM, and microbiota *via* mechanisms of endogenous RNA competition, RNA transcription regulation, protein sponges, and translation regulation (126, 127).

miRNA occupy advantages in the future for early non-invasive diagnosis. Using serum from healthy people and CD patients, an earlier study found miR-19 to have lower expression in the serum of CD patients (23).

## Present Treatment Options for Intestinal Fibrosis and Stricture

A variety of drugs and standardized treatment guidelines are available for IBD, and these measures are confirmed to be effective to relieve inflammation in IBD. However, no anti-fibrotic drug is currently approved, although some present popular anti-inflammation drugs appear effective against fibrosis. When IBD patients develop severe intestinal fibrosis and stricture, the first treatment choice is surgery. However, surgery does not always resolve fibrosis, thus, it persists and continues to develop, and new strictures may appear (128). Therefore, the observation that some of the anti-inflammatory drugs can alleviate fibrosis and stricture to a certain extent, is crucial. **Table 2** shows the present treatment measures in IBD-associated fibrosis and stricture in addition to surgery for resection. Although these current therapeutic options can alleviate the suffering of patients to a certain degree, the preventive rate is low, and surgical methods also have a high or low recurrence rate. This provides grounds to seek more effective treatment options.

**Table 2:** Present treatments in IBD-associated fibrosis.

	Type	Treatment route	Effect	Reference
Drugs	Mesalazine	Oral	Lower endoscopic postoperative recurrences in Mesalazine group compared with placebo groups	(129)
	Azathioprine	Oral	49% of patients are free of rehospitalization in 36 mouths	(130)
	Infliximab	Intravenous injection	No development of new small bowel stenosis; Part of stenosis completely regressed	(27, 131–134)
	Adalimumab	Subcutaneous injection	Effectively prevent the occurrence of small bowel stenosis; keep patients being free of surgery	(135, 136)
	Vedolizumab	Intravenous injection	The patient remains in clinical and endoscopic remission without need for surgical treatment.	(137)
	Thiopurines	Oral	Early use is associated with preventing surgery and the development of fibrostenosis	(138)
Surgery	Endoscopic stricturotomy (ESt)	Endoscopic	Offers comparable surgery-free survival; Avoids a surgical resection for a stricture at a previous ileocolonic anastomosis (ICA); Appears to be effective in treating short ICA strictures with no pre-stenotic proximal dilation in CD patients	(139)
	Stenting	Endoscopic	Effective treatment for strictures relapse; High technical success rate; Risk of adherence of the stent to the mucus membrane of the bowel, perforation, and spontaneous distal migration of the stent	(140)
	Endoscopic balloon dilation (EBD)	Endoscopic	Complete through gastrointestinal endoscopy; Reduce the need for surgery for resection	(141)

MSCs were discovered in the past few years and quickly became a research hotspot in curative and regenerative medicine, with an outstanding performance in tissue regeneration. Considering the admirable therapeutic prospects of MSCs, its application in intestinal fibrosis offers hope for future treatment.

## Therapeutic Prospects of MSCs in Intestinal Fibrosis

### Characteristics of MSCs

MSCs, which possess great self-renewal and multilineage differentiation potential, have been certified to have a great therapeutic effect, including tissue regeneration. Relying on their low antigenicity, MSCs are an expectant hope as a potential therapy in the future. In addition to their wonderful self-renewal and multilineage differentiation abilities, MSCs also possess multipotency with osteogenic, chondrogenic, and adipogenic potentials. All MSCs express similar surface markers such as a cluster of differentiation CD73, CD90, and CD105 and lack the expression of CD14, CD34, CD45, and human leukocyte antigen-DR (HLA-DR) (142).

Since the discovery of MSCs from bone marrow in 1968, a variety of sources have also been confirmed to produce MSCs, like adipose tissue (143), human umbilical cord (144), Wharton’s Jelly (145), placenta (146), among others. These MSCs have been confirmed to have therapeutic potential in both experimental and clinical settings among many diseases including IBD, cardiovascular conditions, Parkinson’s disease, osteoarthritis, diabetes, neurological conditions, wounds, and malignancies (147). Some of the most classic cases of the application of MSCs include certain clinically intractable diseases such as spinal cord injury (148), autoimmune diseases (149), and liver diseases (150). MSCs function not only in tissue regeneration but also in drug delivery. They serve as anti-cancer drug delivery vehicles by loading nanoparticles to be delivered to the tumor microenvironment, producing high transport efficiency (151).

The study of MSCs in IBD has been extensively explored, where MSCs actively ease IBD. For example, umbilical cord MSCs (ucMSCs) are commonly used in disease treatment and their transplantation (from Kunming mice and humans) can effectively protect mice from intestinal injury (152). In addition, ucMSCs can also attenuate colitis through regulating immune cells and associated cytokines. For instance, ucMSCs attenuate IBD by releasing miR148b-5p to inhibit the expression of 15-lox-1 in macrophages (153) and by inhibiting ERK phosphorylation in neutrophils (154). Moreover, clinical trials of MSCs performed on luminal IBD have been proven effective (155). However, there are limitations as the safety and stability data are not absolute, and the effective result is also accompanied by side effects (156). Meanwhile, the number of patients participating in such trials remains small, thus, larger trials are needed in the future.

Exosomes that are secreted by MSCs constitute the classical functional mechanism behind the therapeutic properties of MSCs. As a subtype of extracellular vesicles (EVs), exosomes are lipid vesicles secreted by cells into extracellular space. The other types of EVs are micro-vesicles (MVs)and apoptotic bodies (157). Exosomes are typically 30–150 nm in diameter and recognized through electron microscopy, NTA, and surface markers such as CD9, CD81, and HSP70. Exosomes are secreted by nearly all cells and have been found in plasma, urine, semen, saliva, bronchial fluid, cerebral spinal fluid (CSF), breast milk, serum, amniotic fluid, synovial fluid, tears, lymph, etc (158). They carry different molecules including proteins, nucleic acid, and lipid, which influence their function. When exosomes are transferred to recipient cells, they influence the phenotype of recipient cells, therefore, exosomes are recognized as an important medium for cell-to-cell commutation (159). In the therapeutic application in IBD, exosomal proteins, RNAs, and lipids capably modulate IBD microenvironmental components such as cytokines, chemokines, immune cells, the gut microbiota, and the intestinal mucosal barrier, as part of the mechanism to repair damage and restore intestinal mucosal functions as extensively reviewed by Ocansey et al. (160).

Compared to MSCs, exosomes derived from MSCs or other cells appear to have more desirable unique structural, compositional, and morphological characteristics as well as predominant physiochemical stability and biocompatibility properties, producing enhanced injury repair and disease resolution in animal models (161). The regulatory effect of exosomes in IBD has been extensively investigated in recent years, where MSCs-derived exosomes alleviate colitis through targeting immune cells such as macrophages (162), T cells (163, 164), and neutrophils (154). Exosomes from immune cells, such as macrophages, can also attenuate DSS-induced colitis (165). It is worth noting that EVs from food sources including bovine milk are confirmed to alleviate CD by regulating the immune environment and microbiota (166). This suggests that EVs derived from materials that are easy to obtain could serve as a more practical and useful research direction.

### MSCs Therapy in Intestinal Fibrosis in IBD

**Figure 3:**
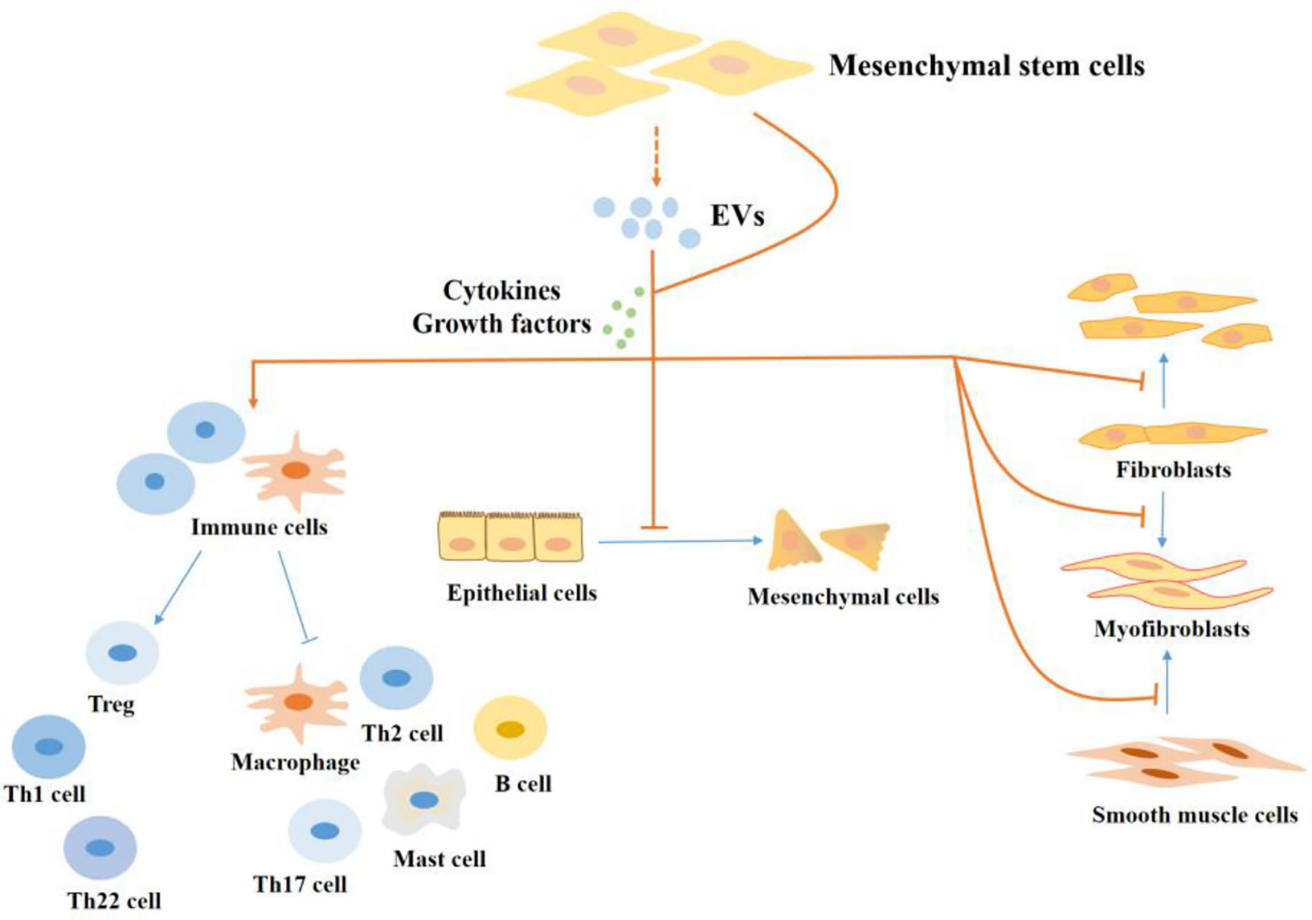
The regulatory effect of MSCs in the progression of fibrosis in IBD. MSCs regulate intestinal fibrosis. MSCs regulate cells in the process of fibrosis by producing cytokines and growth factors or by secreting EVs. MSCs inhibit the proliferation and activation of pro-fibrotic immune cells, like Th17 cells, Th2 cells, M1 macrophages, and mast cells, and promote the production of M2 macrophages and Tregs which inhibit fibrosis. In addition, MSCs inhibit the EMT process and the production of myofibroblasts. EVs, extracellular vesicles; Th, T helper; Treg, Regulatory cells.

Currently, available research on the function and mechanism of MSCs in intestinal fibrosis is severely minimal. However, the few available studies largely present a good prospect of MSCs therapy in intestinal fibrosis, a potential opportunity for both preventing and treating fibrogenesis **Figure 3**. For example, Lei Lian and colleagues found that bone marrow-derived MSCs reduced fibrotic associated activities such as collagen deposition and EMT in the TNBS-induced colitis mice model (167). In another study of colorectal fibrosis, MSCs mediated the downregulation of fibrogenesis *via* controlling ECM turnover. Further investigation revealed that MSCs induced a decreased expression of profibrotic genes and proteins by releasing hepatocyte growth factor (HGF) and tumor necrosis factor-stimulated gene 6 (TSG-6) (168). The anti-fibrotic effects of MSCs through the release of HGF are reported in other tissue injuries including liver fibrosis (169). and lung fibrosis (170).

Both allogeneic and autologous MSCs transplantation is safe and consequently represents a treatment option for fibrosing diseases, fistulizing colitis like CD, and refractory connective tissue diseases, as they are non-immunogenic (171, 172). Moreover, systemic administration of MSCs for the treatment of refractory irradiation-induced colitis was safe and effective on pain, diarrhea, hemorrhage, inflammation, and fistulization accompanied by regulation of the lymphocyte subsets towards an elevated Tregs cell and a reduction of activated effector T cells (173, 174). A study revealed that both i.v. infusion and i.m. injunction of MSCs after anal sphincter injury in rats resulted in a marked decrease in fibrosis and scar tissue compared with PBS-treated groups (175).

The EVs derived from MSCs affect the development of intestinal fibrosis through their cargoes, including proteins and RNAs. A study by Jia Yang and colleagues demonstrated that micro-vesicles containing miR-200b attenuate colitis-associated fibrosis by preventing EMT (27). A recent study of experimental CD examined the effect of MSCs engineered to overexpress hypoxia-inducible factor 1-alpha and telomerase (MSC-T-HIF) and conditioned with pro-inflammatory stimuli to release EVs (EVMSC-T-HIFC) on fibrosis and inflammatory response of activated endothelium. The authors found that in addition to dampening inflammation, the EVMSC-T-HIFC prevented myofibroblast differentiation of TGF-β-treated fibroblasts (176). In a similar study, paracrine factors derived from MSCs were shown to protect against lung fibrosis in terms of fibrotic scores, collagen deposition, inflammation, and cell apoptosis (177). It is also exciting that MSC treatment is confirmed to be effective and safe in a clinical trial on idiopathic pulmonary fibrosis patients (178). The administration of culture supernatant of MSCs significantly reduced the degree of luminal stricture in the rectum and attenuated myofibroblast activation and hypertrophy of the muscularis propria in pigs (179). In clinical trial, autologous bone marrow-derived MSCs was confirmed to control the inflammation in IBD, especially in inhibiting TNF-α production (171). In a phase 2 study, administration of allogeneic MSCs reduced CD activity index and CD endoscopic index of severity scores in patients with luminal CD refractory to biologic therapy (180). Although these clinical trials did not examine fibrotic proliferation and stenosis, the results provide an important proof-of-principal and intention of follow-up research on fibrosis, considering the relationship between inflammation and fibrosis.

In addition, an anal fistula is a common complication of CD which mainly occurs around the anal rather than intestinal lumen. However, the development of the fistula is closely related to fibrosis. Studies show that MSCs are an optional treatment for fistula. Allogeneic adipose-derived MSCs can reduce the occurrence of fistula in CD patients (181). Moreover, injection of autologous adipose-derived MSCs is safe and could completely heal 57% of patients with fistulas and reduce secretion in part of remaining patients (182). Furthermore, allogeneic and autologous adipose-derived MSCs have shown advantageous results for fistula treatment in long-term clinical trials, and are proven to be safe (183, 184).

As expected of every research field, the hope of MSCs and related EVs therapy in intestinal fibrosis is to translate the outcome into clinical application. Although some clues have been shown regarding the possibility of MSCs application in intestinal fibrosis, the evidence is far from enough. There is still a long way to go for laboratory research to transform into clinical applications, as several questions remain unanswered and mechanisms largely unclear.

Concerning other challenges in this field of study, previous research on fibrosis used different animal models, usually mice or rats, which are confronted with many limitations. For better experimental outcomes and subsequent clinical application, there is the need to use models closer to the details of the disease. Intestinal organoids have been widely used in intestinal research recently and have also been reported in the research of intestinal fibrosis (94). As a more three-dimensional and more specific experimental model, it can also be used in future research.

### The Function of MSCs in Other Tissue Fibrosis

#### Liver Fibrosis

Liver fibrosis which is triggered by viral or metabolic chronic liver diseases is one of the common fibrosis in the clinical setting and has the risk of transition to cancer (185). Due to the admirable effect of MSCs on other diseases, researchers have set to explore the function of liver fibrosis. Concerning immune cells, macrophage has been proven to play a significant role in the progression of liver fibrosis. Xiao-Yu Luo et al. reported that the transplanted BM-MSC can increase the M2/M1 macrophage ratio through migrating to injury liver location, and the action, in turn, affects hepatic stellate cells (HSCs) apoptosis (186). Ly6Chi/lo macrophages are two different types of macrophages in the liver. Ly6Chi is highly inflammatory and fibrotic while Ly6Clo could alternatively decrease liver inflammation and fibrosis through secreting certain cytokines. A study confirmed that BM-MSCs ameliorate liver fibrosis by regulating Ly6Chi/Ly6Clo conversion and preventing Ly6Chi recruitment (187). Apart from macrophage, MSCs also promote liver regeneration through regulating neutrophils (188) and T cells (189). The cross-talk between MSCs and Tregs is crucially important for the attenuation of acute liver injury.

Engineered MSC possesses an enhanced regulatory effect in inhibiting and reversing liver fibrosis. IC-2 engineered BM-MSC was proven to have the potential to relieve liver fibrosis (190). Just as indicated earlier, MSCs functionally alleviate liver fibrosis by producing cytokines or factors, directly acting on target cells, and secreting EVs. Earlier research shows that BM-MSC-derived exosomes could potently relieve fibrotic change in the CCI4 rat model and protect the function of the liver. It is worth noting that many comparative studies report that the effect of BM-MSC exosomes is better than BM-MSC itself (191). Li yang Dong and colleagues confirmed that hucMSC-EV was able to effectively ameliorate liver fibrosis in rat models by inhibiting HSC activation (192). In CCI4 rat models, EVs from amnion-derived MSCs were also capable of targeting HSCs activation to relieve liver fibrosis (193). In recent research, the authors combined MSCs with Kampo medicine Juzentaihoto (JTT), which is dried and powdered from 10 crude drugs, and used it for liver fibrosis therapy in animal models. The results showed that the combination therapy attenuated liver fibrosis by the JTT increasing the CD4+/CD8+ratio while MSCs promoted the transition of inflammatory macrophages to anti-inflammatory macrophages (194).

#### Kidney Fibrosis

Kidney fibrosis is the final outcome in the progression of certain kidney diseases, especially chronic kidney diseases (CKD). Recent reports show that hucMSCs effectively ameliorate renal fibrosis in DN rats, including decreasing fibrotic molecules expression and restoring tissue integrity. Meanwhile, hucMSCs would depress TGF-β expression, which has been widely confirmed to be a key cytokine in tissue fibrosis and secret anti-fibrotic molecule in the tubular epithelial cells (195). Another research reported that the transplantation of BM-MSCs induced anti-fibrotic events in rats by decreasing collagen production and myofibroblast accumulation. Besides, BM-MSCs could also regulate non-coding RNAs, such as miRNAs to influence downstream proteins for easing fibrosis (196).

#### Pulmonary Fibrosis

Pulmonary fibrosis is a type of lung chronic diseases complication, with a high global incidence. Studies show that the incidence of pulmonary fibrosis in Europe and North America is estimated to range between 2.8 and 18 cases per 100000 people per year (196). However, the major health and safety incidents- the epidemic of COVID-19 pushed it to become a more meaningful need for finding anti-fibrotic therapy (197). In experimental models, MSCs can act on immune cells to regulate lung fibrosis. In a clinical trial, idiopathic pulmonary fibrosis patients who received doses of allogeneic MSCs showed greater performance in lung function examinations compared with patients with placebo (178). In addition, MSCs-EVs have also shown positive implications in lung fibrosis. Studies show EVs from human BM-MSCs could prevent and alleviate pulmonary fibrosis by changing the phenotype of monocyte (198). Regardless, MSCs may have a profibrogenic function. As a type of stem cell which owns multilineage differentiation, MSCs might have the chance to differentiate to myofibroblast under certain stimuli on the process of fibrosis prevention and therapy. For example, a study found that BM-MSC could accelerate lung fibrosis through a transform to myofibroblast (199). This calls for more investigations in establishing a stable condition in which MSCs and their secretory products could induce and sustain anti-fibrotic effect to a period necessary to produce the desired outcome.

**Figure 4:**
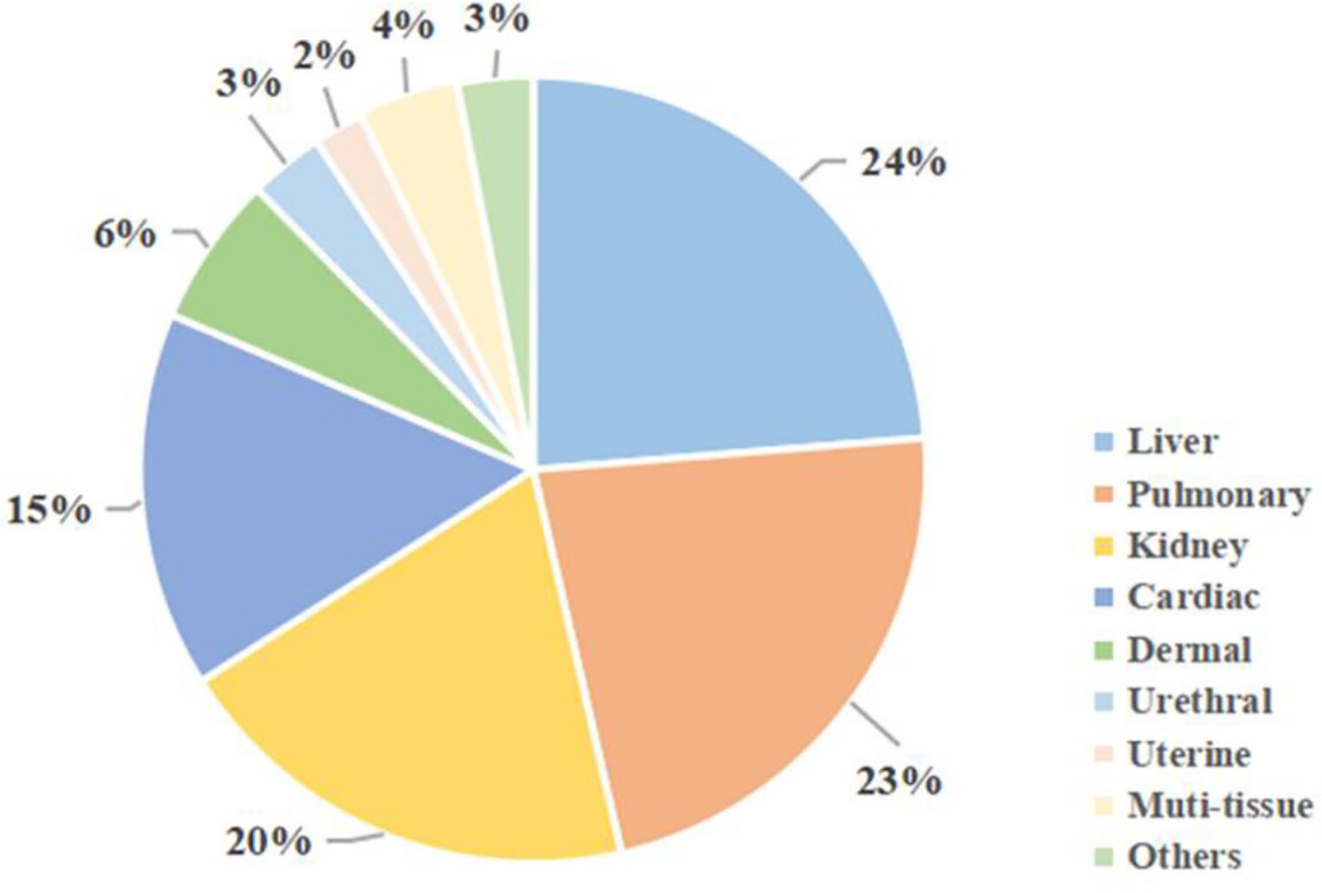
MSCs studies on different tissue fibrosis in recent 5 years. Using the keywords “fibrosis” and “mesenchymal stem cells”, as well as specific organs, a search was conducted on the PubMed online library. Results were restricted to studies published from 2017 to 2021.

Apart from the three tissues above, MSCs could have a profibrogenic function in other tissues. **Figure 4** shows MSCs-related studies on different tissues in recent 5 years, from 2017 to 2021.

## Conclusion

Intestinal fibrosis has a high incidence rate in the course of IBD and there is no ideal treatment solution currently. Fortunately, studies have confirmed that MSCs and their secretory products such as exosomes can alleviate fibrosis by inhibiting the EMT process and reducing collagen deposition. Although studies on MSCs application in intestinal fibrosis in IBD are woefully low, by considering the promising role of MSCs in collective studies on fibrosis of organs, we have reasons to believe that it can also play an effective role in intestinal fibrosis. The mechanisms involved still need further exploration.

## Author Contributions

YW and BH determined the topic of the article, proposed a program, and wrote the article. TJ summarized and drew diagram of the mechanism. DO modified the language of the article. JJ and FM collected literature and guided the article and gave opinions. All authors have read and approved the final manuscript.

## Funding

This work was supported by the Science and Technology Innovation Fund Project of Zhenjiang City (grant no. SH2021066) and the Technology Project of Zhangjiagang (ZKCXY2106).

## Conflict of Interest

The authors declare that the research was conducted in the absence of any commercial or financial relationships that could be construed as a potential conflict of interest.

## Publisher’s Note

All claims expressed in this article are solely those of the authors and do not necessarily represent those of their affiliated organizations, or those of the publisher, the editors and the reviewers. Any product that may be evaluated in this article, or claim that may be made by its manufacturer, is not guaranteed or endorsed by the publisher.
